# miR-765 induces angiogenesis by inhibiting dipeptidyl peptidase 4 and increasing fibroblast growth factor 2

**DOI:** 10.1016/j.bbadva.2026.100193

**Published:** 2026-04-29

**Authors:** Koji Ueno, Hiroshi Kurazumi, Junichi Murakami, Takahiro Mizoguchi, Ryo Suzuki, Toshiki Tanaka, Kimikazu Hamano

**Affiliations:** a Department of Surgery and Clinical Science, Graduate School of Medicine, Yamaguchi University, Ube, Yamaguchi, Japan; b Division of Advanced Cell Therapy, Research Institute for Cell Design Medical Science, Yamaguchi University, Ube, Yamaguchi, Japan

**Keywords:** Extracellular vesicles, microRNA, Angiogenesis

## Abstract

Given that extracellular vesicles (EVs) secreted from stem cells contain angiogenic microRNAs (miRNAs), these EVs show equivalent angiogenic therapeutic effect to cell transplantation. This study aimed to identify the angiogenic miRNAs from several miRNAs involved in EVs secreted from stem cells. In human dental pulp stem cells (hDPSCs) under hypoxic culture, vascular endothelial growth factor (VEGF) involved in EVs increased. We hypothesized that angiogenic miRNAs increase in EVs that are secreted from hDPSCs under hypoxic culture compared with those under normoxic culture. In the first screening, the expression levels of miRNAs involved in EVs that were secreted from hDPSCs cultured under hypoxic and normoxic conditions were analyzed using miRNA array. In the second screening, 12 miRNAs were individually involved in EVs, and the growth of human aortic endothelial cells was assayed. In the third screening, miRNA-encapsulated EVs were injected in BALB/c mouse hindlimb ischemia model, followed by angiogenesis evaluation by blood flow analysis. Results showed that miR-765 is an angiogenic miRNA, targeting the 3′ untranslated region of dipeptidyl peptidase 4 (DPP4) and upregulating fibroblast growth factor 2 (FGF2) in vitro and in vivo. In conclusion, as an angiogenesis mechanism, miR-765 increased FGF2 expression levels by inhibiting DPP4.

## Highlights

• EVs secreted from human dental pulp stem cells exhibited an angiogenic effect.
• miR-765 is contained in EVs secreted from human dental pulp stem cells.
• miR-765–encapsulated EVs improve the mRNA expression levels of angiogenic genes.
• miR-765 increases FGF2 expression levels through DPP4 inhibition.

## Graphical abstract

**Figure:**
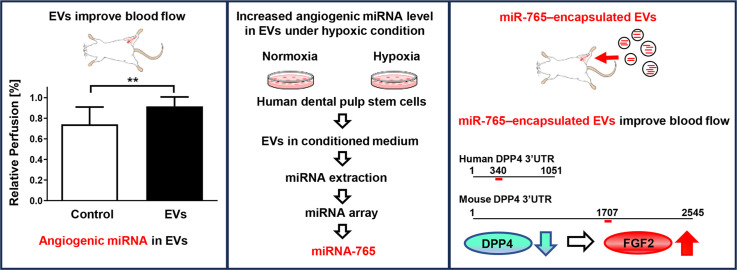

## Introduction

In the US, Europe, and Japan, roughly 6.5 million patients have been diagnosed with critical limb ischemia (CLI) [1]. CLI is the advanced stage of peripheral artery disease (PAD), particularly atherosclerotic occlusive disease, which exhibits insufficient blood flow resulting from arterial narrowing [2,3]. Its first treatment choice is revascularization by endovascular therapy and surgical bypass [4]. Revascularization surgery has been performed in 50% of patients with CLI, and amputation in 25% [5]. However, 35%–40% of patients with CLI lose legs, and 20% die within 6 months from initial diagnosis [5,6]. Approximately 45%–50% retain both legs, while 25%–30% lose one leg; unfortunately, 25% die within 1 year from initial diagnosis [6,7]. To reduce the number of amputation cases, we need a novel therapy that induces blood flow in ischemic limbs.

Cell transplantation therapy has been researched for inducing blood flow in ischemic limbs [[8], [9], [10]]. We transplanted autologous bone marrow cells in patients with CLI [11]. To reduce the invasiveness of cell isolation for these patients, we changed the transplanted cells from bone marrow cells to peripheral blood mononuclear cells (PBMNCs); a hypoxic preconditioning method was also developed to enhance the effect of blood flow and survival rate of transplanted cells [[12], [13], [14]]. In brief, we transplanted autologous PBMNCs that received a hypoxic preconditioning method in patients with CLI [15]. Angiogenesis after stem cell transplantation is a paracrine effect induced by extracellular vesicles (EVs) that are secreted from transplanted stem cells [[16], [17], [18], [19], [20], [21], [22], [23], [24]]. Although typical EVs are exosomes [25], they are currently classified into small EVs (sEVs), medium EVs (mEVs), and large EVs (lEVs), based on their size [26]. EVs contain microRNAs (miRNAs) (e.g., miR-126-3p, miR-199b-5p, miR-24, and miR-125a-5p) that are reportedly essential in angiogenesis [[17], [18], [19], [20]]. Angiogenesis induced by EVs in the mouse hindlimb ischemia model has been reported, focusing on exosomes secreted from stem cells and miRNAs contained in exosomes to elucidate its mechanism. These reports suggest that encapsulating angiogenic miRNAs in EVs is essential for angiogenesis therapy by EVs.

We hypothesized that cells incubated by a hypoxic preconditioning method secrete EVs containing angiogenic miRNAs, given that such cells secrete a larger amount of vascular endothelial growth factor (VEGF) in the culture medium than those cells incubated with normal oxygen concentration [[27], [28], [29], [30]]. In the present study, human dental pulp stem cells (hDPSCs) were incubated under the hypoxic preconditioning method, and miRNAs encapsulated in EVs were analyzed to identify angiogenic miRNAs. After some screening experiments, our results suggest that miR-765 is an angiogenic miRNA.

## Methods

### Animals

The Institutional Animal Care and Use Committee of Yamaguchi University approved all our animal procedures (#31–101). The methods conformed to the approved guidelines and the ARRIVE guidelines. Male BALB/c mice were purchased from Japan SLC, Inc. (Hamamatsu, Shizuoka, Japan). Five mice were housed in the same cage (mouse polycarbonate cage, #TM-PC-5-I(1), Tokiwa, Tokyo, Japan). Fir wood shavings were used as beddings. These mice were bred in a temperature-, humidity-, and light-controlled sub-specific pathogen free room (22 ± 2°C, 70% ± 20%, and 12 h light/dark cycles, respectively) at the Institute of Life Science and Medicine Yamaguchi University. Food and water were provided ad libitum. The mice were randomly divided into groups. Anesthesia was maintained with 1.5%–2% isoflurane (MSD Animal Health, Tokyo, Japan).

### Cells

We purchased hDPSCs from Lonza (#PT-5025; Basel, Switzerland) and cultured them in CiMS™-BM (#A2G00P05C, Cell Science & Technology Institute, Inc., Sendai, Miyagi, Japan) supplemented with CiMS™-sAF (#A2G20P1CC, Cell Science & Technology Institute, Inc.) or KBM ADSC-4 (#16,030,044, Kohjin Bio Co., Ltd., Sakado, Saitama, Japan). The 293 T cells (#RCB2202) were provided by the RIKEN BRC through the National Bio-Resource Project of the MEXT/AMED in Japan and cultured in Dulbecco’s modified Eagle medium (DMEM; #11,995–065, Thermo Fisher Scientific, Waltham, Massachusetts, USA) supplemented with 10% exosome-depleted fetal bovine serum (FBS; #A2720801, Thermo Fisher Scientific) or HE100 medium (Gmep, Inc., Kurume, Fukuoka, Japan) with L-glutamine. Furthermore, we purchased HAoEc from TaKaRa Bio, Inc. (#D10039, Kusatsu, Shiga, Japan) and cultured them in Endothelial Cell Growth Medium MV 2 (Ready-to-use) (#D12020, TaKaRa Bio Inc.) We purchased Human skeletal muscle myoblasts (HSMMs; #CC-2580, Lonza) were cultured in SkGM™-2 Skeletal Muscle Cell Growth Medium-2 BulletKit™ (#CC-3245, Lonza).

### Measurement of growth factors and cytokines

For 3 days, hDPSCs (2 × 10^5^ cells/mL) were cultured under normoxic conditions (37°C in 5% carbon dioxide (CO_2_) and atmospheric oxygen concentrations). We centrifuged the conditioned medium at 3000 rpm for 3 min at 4°C and stored the supernatant at −80°C. EVs in the supernatant were collected using total exosome isolation reagent (from cell culture media) (#4478,359, ThermoFisherScientific). To isolate proteins in EVs, we dissolved EV pellets by using an exosome resuspension buffer contained in Total Exosome RNA and Protein Isolation Kit (#4478,545, Thermo Fisher Scientific) and stored them at −80°C after vortex. Moreover, the concentrations of growth factors and cytokines in the cell culture supernatant and EVs were measured using VEGF (# DVE00), angiopoietin-1 (# DANG10), HGF (# DHG00), IL-4 (# D4050), PDGF-FF (# DBB00), and FGF2 (# HSFB00D) ELISA kits (R&D Systems, Inc., Minneapolis, Minnesota, USA) according to the manufacturer’s instructions.

### Blood flow improvement effect after EV administration in the hindlimb ischemia model

We cultured hDPSCs in CiMSTM-BM supplemented with CiMSTM-sAF for 5 days under normoxic conditions. EVs were then isolated from the conditioned medium by using the total exosome isolation reagent (from cell culture media) and resuspended in phosphate-buffered saline solution (PBS). In addition, the protein concentration was measured using the MicroBCA Protein Assay kit (#23,235, Thermo Fisher Scientific). Next, the left arteriovenous of BALB/c mice was ligated in one place. The next day, 30 µg of EVs were injected in two places in the femoral muscle. We then analyzed the blood flow via a laser speckle perfusion imaging system (OMEGA ZONE, Omega Wave, Tokyo, Japan). The value of the ischemic leg was indicated as 1 for the healthy leg.

### Comparison of cell proliferation and VEGF concentration in EVs depending on the medium and culture method by oxygen concentration

We seeded hDPSCs (1 × 10^3^ cells/100 mL/well) in 96-well plates by using KBM ADSC-4 and cultured them under normoxic conditions (37 °C, 5% CO_2_, atmospheric oxygen concentration) or hypoxic conditions (33 °C, 5% CO_2_, 2% O_2_). Cell proliferation was analyzed using the CellTiter 96® AQueous One Solution Cell Proliferation Assay (MTS) (#G3580, Promega, Madison, Wisconsin, USA) at 1, 3, 5, and 7 days after cell seeding. The 490 nm absorbance was measured using ARVO X4 (PerkinElmer, Boston, Massachusetts, USA). We again cultured hDPSCs under normoxic and hypoxic conditions for 7 days. Protein concentrations in EVs were isolated, and the VEGF concentration was measured using the abovementioned method. The EV concentration was measured using a CD9/CD63 exosome ELISA kit (#EXH0102EL, Cosmo Bio Co., Ltd., Tokyo, Japan).

### miRNA extract and array analysis

We seeded hDPSCs (2 × 10^5^ cells/10 mL) into 10 cm dish in KBM ADSC-4 and cultured 10 dishes of each for 7 days under normoxic and hypoxic conditions. The conditioned medium was concentrated using Amicon Ultra-15(#UFC901024, Merck Millipore Ltd., Burlington, Massachusetts, USA). Using the total exosome isolation reagent (from cell culture media), we made pellets from the concentrated liquid of the conditioned medium. Next, microRNAs were isolated from the pellets by using the miRNeasy Mini Kit (#217,004, Qiagen Venlo, Netherlands). The miRNA expression levels were analyzed using 3D-Gene® miRNA oligochips (Toray Industries, Inc., Kamakura, Kanagawa, Japan) (GEO accession: GSE232178 and Supplementary Data).

### Plasmids expressing miRNAs

To construct the plasmids expressing the 12 miRNAs, we amplified the 293 T cell genome with Ex Taq (TaKaRa Bio, Inc.) and primers and ligated the amplified DNA to a T-vector pMD20 (#3270, TaKaRa Bio Inc.). Primers were designed with reference to the human chromosome region. DNA fragments were subcloned from the T-vector pMD20 into the restriction enzymes’ site of the pmR-mCherry vector (#Z2542N, TaKaRa Bio Inc). Details are presented in Supplementary Table 1.

### Preparation of miRNA-encapsulated EVs

The 293 T cells (2 × 10^5^ cells/2 mL/well) were seeded in six-well plates by HE100 medium with L-glutamine and then incubated overnight. Next, the cells were transfected with 2 µg of plasmid by using 2 µL of X-tremeGENE HP DNA transfection reagent (Sigma-Aldrich Japan K.K., Tokyo, Japan) and incubated for 24 h. After removing the culture medium from the wells, we applied 2 mL/well of fresh HE100 medium supplemented with L-glutamine to the wells. Three days after, we collected the culture medium and centrifuged it at 3000 rpm for 4 min at 4°C. Subsequently, the supernatant was applied to a qEV original 70 column (Meiwafosis Co., Ltd., Tokyo Japan) to isolate the EVs. The EV concentration was then measured using a CD9/CD63 exosome ELISA kit (Cosmo Bio Co., Ltd.).

### Cell proliferation assay after adding EVs

We seeded HAoEc (1 × 10^3^ cells/100 µL/well) into 96-well plates, adjusted the EVs to 750 pg/mL in PBS, and added 100 µL of the solution to each well. Next, we incubated the HAoEc for 7 days, followed by cell proliferation assay using MTS.

### Preparation of miRNA-containing EVs for screening using the hindlimb ischemia model

We seeded 293 T cells in six-well plates by DMEM supplemented with 5% exosome-depleted FBS. These cells were transfected with 2 µg of plasmid by using 2 µL of X-tremeGENE HP DNA transfection reagent and then incubated overnight. After removing the culture medium from the wells, we applied 2 mL/well of fresh DMEM supplemented with 5% exosome-depleted FBS to the wells. The culture medium was collected after 5 days. The EVs in the culture medium were isolated using the total exosome isolation reagent (from cell culture media). The pellets were then dissolved with 400 µL of PBS. Furthermore, the left arteriovenous of 8-week-old BALB/c mice was ligated in one place. The next day, 100 µL of PBS including EVs were injected in two places in the femoral muscle. We subsequently analyzed the blood flow via a laser speckle perfusion imaging system (OMEGA ZONE) (6 mice per group).

### Western blotting for EVs

EVs (1000 pg/300 µL) were pelletized using the total exosome isolation reagent. These pellets were dissolved with 50 µL of 4 × Laemmli sample buffer (#1610,747, Bio-Rad, Hercules, California, USA). Subsequently, 10 µL of each sample (200 pg of EVs) was applied to each well and subjected to western blotting using a CD9 monoclonal antibody (Ts9; 1:500; #10626D, Thermo Fisher Scientific), a CD81 monoclonal antibody (M38; 1:500; #10630D, Thermo Fisher Scientific), a GM130 (D6B1) XP® rabbit monoclonal antibody (1:500; #12,480, Cell Signaling Technology, Inc., Danvers, Massachusetts, USA), a goat antimouse Ig/HRP (affinity isolated) antibody #P0447; Dako, Glostrup, Denmark), and a goat antirabbit Ig/HRP (affinity isolated) antibody (1:2000; #P0448, Dako). To visualize the extracted proteins, we used the ECL™ prime western blotting detection reagent (#RPN2232, Cytiva, Marlborough, Massachusetts, USA). The images were then detected using Amersham™ Imager 600 (Cytiva). We used Precision Plus Protein™ Dual Color Standards as a marker (#1610,374, Bio-Rad).

### EV size distribution and morphology

The EV size distribution was analyzed using LM10 NanoSight (Malvern Instruments, Malvern, UK). The relevant results are shown by standard error (n = 5). We used a transmission electron micrograph to observe EV morphology.

### Analysis of miRNA expression levels using quantitative polymerase chain reaction (qPCR)

The miRNAs were extracted from cells, and 1000 pg of EV pellets were made using the abovementioned method with the use of miRNeasy mini kits and the total exosome isolation reagent. We also synthesized cDNAs from miRNAs by using the TaqMan™ advanced miRNA cDNA synthesis kit (#A28007, Thermo Fisher Scientific) and conducted qPCR by using the cDNAs with a TaqMan™ fast advanced master mix (#4444,557, Thermo Fisher Scientific) and the following TaqMan advanced miRNA assays (Thermo Fisher Scientific): hsa-miR-186-5p (477940_mir) which was selected as an internal control referring to manual instruction manual of TaqMan advanced miRNA assays (Thermo Fisher Scientific) and hsa-miR-756 (479173_mir). Thereafter, we performed qPCR on a StepOnePlus instrument (Thermo Fisher Scientific), employing the following qPCR parameters for cycling: 95°C for 20 s, 40 cycles of PCR at 95°C for 1 s, and 60°C for 20 s. All reactions were undertaken thrice in 20 µL reaction volumes. The miRNA expression levels were determined using the 2^–D^CT method.

### Analysis of the mRNA expression levels after injecting miR-765-containing EVs in hindlimb ischemia model mice

Scramble and miR-765 mimics were synthesized by Ajinomoto Bio-Pharma Services, GeneDesign Inc. (Osaka, Japan). We seeded 293 T cells in a 10 cm dish (2 × 10^6^ cells/dish) by using 10 mL of HE100 medium supplemented with L-glutamine. The next day, these cells were transfected with 600 pmol of miRNA mimics by using 25 µL of the RNAiMAX transfection reagent (#13,778,075, Thermo Fisher Scientific) and then incubated for 24 h. After removing the culture medium from the dishes, we applied 10 mL/dish of fresh HE100 medium supplemented with L-glutamine to the dishes. After 3 days, we collected the culture medium, which was then concentrated with Amicon Ultra-15 (#UFC901024, Merck Millipore Ltd., Japan Headquarters, Tokyo, Japan). This concentrated solution was applied to a qEV original 70 column (Meiwafosis Co., Ltd., Tokyo Japan) to isolate the EVs. The EV concentration was subsequently measured using a CD9/CD63 exosome ELISA kit (Cosmo Bio Co., Ltd.). The left femoral artery of 8-week-old BALB/c mice was ligated in one place, and after 4 days, 100 pg of EVs were injected in two places in the femoral muscle. Two days after, the left thigh muscles were extracted and immersed in RNAlater™ stabilization solution (#AM7020, Thermo Fisher Scientific) and then stored at 4°C. Moreover, total RNAs were extracted using a RNeasy Mini Kit (#74,104, Qiagen) and then reverse-transcribed into single-stranded cDNA by using PrimeScript™ RT reagent kits (Perfect Real Time; # RR037A, TaKaRa Bio, Inc.). On a StepOnePlus instrument (Thermo Fisher Scientific), qPCR was performed using cDNAs with SYBR™ select master mix (#4472,918, Thermo Fisher Scientific). Supplementary Table 2 summarizes the primer sequences used. The qPCR parameters for cycling were as follows: 50°C for 2 min, followed by 95°C for 2 min, 40 cycles of PCR at 95°C for 3 s, and 60°C for 30 s. All reactions were performed thrice in 10 µL reaction volumes. The mRNA expression levels were determined using the 2^–D^CT method.

### 3′UTR luciferase reporter assay

A pmirGLO dual-luciferase miRNA target expression vector (#E1330, Promega) was used for the 3′UTR luciferase assays. The 3′UTRs of the human and mouse DPP4 sequences targeted by miR-765 were predicted using the RNA22 v2 miRNA target detection software (https://cm.jefferson.edu/rna22/Interactive/). Supplementary Tables 3 and 4 summarize the oligo sequences used for constructing 3′UTR plasmids for human and mouse DPP4. The plasmids for the 3′UTR luciferase assays were constructed as described previously [31]. For the 3′UTR luciferase assay, 293 T cells were seeded to 48-well plates (2 × 10^4^ cells in 0.2 mL/well) by DMEM supplemented with 10% FBS (#10,437,028, Thermo Fisher Scientific) and cultured at 37°C in 5% CO_2_ overnight. Next, they were transfected with 40 pmol of the miR-765 mimic and 200 ng of pmirGLO dual-luciferase miRNA target expression vectors with wild-type or mutated target sequences by using 0.67 µL of lipofectamine 2000 (Thermo Fisher Scientific) in each well of a 48-well plate. After 2 days of transfection, the luciferase assay was performed using the Dual-Luciferase® Reporter Assay System (#E1910, Promega). The Genetic Modification Safety Committee of Yamaguchi University approved our recombinant DNA study (#J20003).

### Western blotting for total protein and membrane protein

At 3 days after transfection, we extracted protein from cells. In extracting total protein, cells were dissolved in extraction buffer containing 10 mM Tris pH 7.4, 0.5 mM ethylenediamine tetraacetic acid, 0.5 mM egtazic acid, 1% Triton X-100, and protease inhibitor, followed by centrifugation at 13,000 rpm for 20 min at 4°C. We collected the supernatant as the total protein. As for extracting membrane protein, cells were resuspended in extraction buffer containing 10 mM Tris pH 7.4, 1% sodium dodecyl sulfate, and 100 mM sodium orthovanadate, followed by incubation at 95°C for 20 min and centrifugation at 17,000 g for 10 min at 20°C. We then collected the supernatant as the membrane protein. Furthermore, 20 µg of each sample was applied to each well and subjected to western blotting using a CD26 polyclonal antibody (1:1000; #PA5-120,281, Thermo Fisher Scientific), a β-actin (13E5) rabbit monoclonal antibody (1:1000; #4970, Cell Signaling Technology, Inc.), an anti-FGF2 (EPR20145-219) antibody (1:1000; #ab208687, Abcam, Cambridge, UK), an alpha/beta-tubulin antibody (1:1000; #2148, Cell Signaling Technology, Inc.), and a goat antirabbit Ig/HRP (affinity isolated) antibody (1:2000; #P0448, Dako).

### Establishment of DPP4-knockdown 293 T cells, HAoECs and HSMM lines

Knockdown cell lines were constructed using a shRNA expression lentivector as previously reported [32]. The target sequences and oligo sequences used for the construction of the plasmids are listed in Supplementary Table 6. HAoECs and HSMM were incubated with pseudoviral particles and TransDux MAX lentivirus transduction enhancer (#LV860A-1; SBI) and cultured with puromycin dihydrochloride (#A1113803; Thermo Fisher Scientific), while 293 T cells were incubated with pseudoviral particles and polybrane (#H9268; Sigma-Aldrich), and cultured with puromycin dihydrochloride (#A1113803; Thermo Fisher Scientific). Western blotting and qPCR analysis were carried out as described above.

### Statistical analysis

All statistical data were analyzed using the GraphPad Prism 8 software (GraphPad Software, USA). A P-value of <0.05 was considered statistically significant: *P < 0.05; **P < 0.01; ***P < 0.001, and ****P < 0.0001. Additionally, bars represent means with SDs.

## Results

### EVs secreted from hDPSCs showed an angiogenic effect

**Fig. 1:**
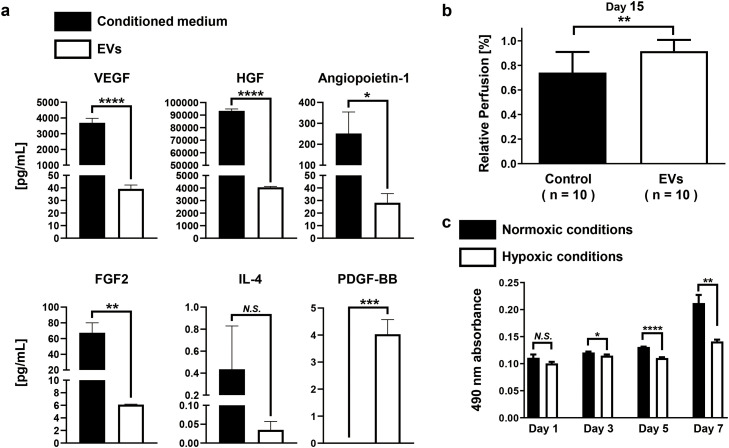
Characteristics of extracellular vesicles (EVs) secreted from human dental pulp stem cells (hDPSCs). a: Comparative analysis of the concentration of growth factors in the conditioned medium and those in EVs secreted from hDPSCs cultured in CiMS™-BM for 3 days under normoxic conditions (37 °C, 5% CO_2_, atmospheric oxygen concentration). The concentration was measured by enzyme-linked immunosorbent assay (ELISA) (The sample size was 3 in each group; unpaired t-test). b: Blood flow was improved in the hindlimb ischemia model after injecting EVs at 1 day after ligation. Blood flow was assessed at 15 days after ligation, and the value of the ischemic leg was indicated as 1 for healthy leg (n = 10/group; unpaired *t*-test). c: The hDPSCs were incubated in KBM ADSC-4 for 7 days under normoxic conditions (37 °C, 5% CO_2_, atmospheric oxygen concentration) or hypoxic conditions (33 °C, 5% CO_2_, 2% O_2_). Hypoxic conditions inhibited hDPSC proliferation. Cell proliferation was assayed using MTS (The sample size was 3 in each group; unpaired t-test). CO_2,_ carbon dioxide; FGF-2, fibroblast growth factor 2; VEGF, vascular endothelial growth factor; HGF, hepatocyte growth factor; IL-4, interleukin 4; O_2_, oxygen; PDGF-BB, platelet-derived growth factor (PDGF) with two B subunits.

To recognize whether EVs contain growth factors, hDPSCs were cultured with CiMS™-BM for 3 days under normoxic conditions and measured the concentration of growth factors in the conditioned medium and EVs through enzyme-linked immunosorbent assay (ELISA) (Fig. 1a). VEGF, angiopoietin-1, hepatocyte growth factor (HGF), and fibroblast growth factor 2 (FGF2) secreted in the conditioned medium from hDPSCs had significantly higher concentrations than the growth factors contained in EVs secreted from hDPSCs; conversely, the concentration of platelet-derived growth factor (PDGF) with two B subunits (PDGF-BB) was significantly larger in EVs than in the conditioned medium. To determine whether EVs secreted from hDPSCs have an angiogenic effect, we injected EVs in the mouse hindlimb ischemia model and analyzed the blood flow 15 days after EV injection (Supplementary Figure 4). Blood flow improved in the ischemic leg where EVs were injected compared with the control (Fig. 1b). To assess the effect of hypoxic conditioning methods, hDPSCs were cultured with KBM ADSC-4 or CiMS™-BM under normoxic and hypoxic conditions. The concentration of VEGF secreted in the conditioned medium from hDPSCs in both medium under hypoxic conditions was higher than that under normoxic conditions (Supplementary Figure 5a). Cultivation in ADSC-4 resulted in a higher VEGF content per unit of EVs as compared to CiMS™-BM (Supplementary Figure 5b). Cell proliferation in KBM ADSC-4 was significantly slower in hypoxic conditions compared with that in normoxic conditions (Fig. 1c).

### Angiogenic miR-765 is included in EVs secreted from hDPSCs with hypoxic culture

**Fig. 2:**
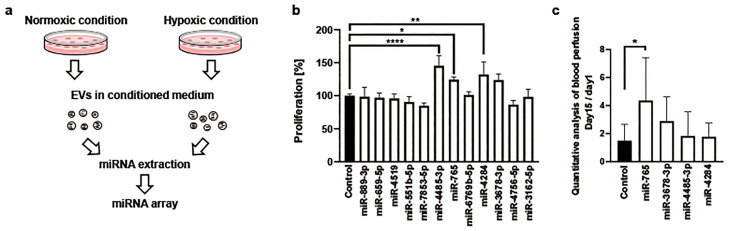
Screening methods for identifying angiogenic microRNAs (miRNAs). a: First screening. The expression levels of miRNA in extracellular vesicles (EVs) were determined by array analysis. Human dental pulp stem cells (hDPSCs) were incubated in KBM ADSC-4 for 7 days under normoxic conditions (37 °C, 5% CO_2_, atmospheric oxygen concentration) or hypoxic conditions (33 °C, 5% CO_2_, 2% O_2_), and miRNAs were extracted from EVs secreted from hDPSCs. b: Second screening. Cell proliferation was assayed in human aortic endothelial cells after adding EVs containing miRNAs was assayed (n = 3/group; Dunnett’s multiple comparisons test). c: Third screening. Blood flow was analyzed in the mouse hindlimb ischemia model after injecting EVs containing miRNAs (n = 6/group; Dunnett’s multiple comparisons test).

From this result, we hypothesized that by incubating hDPSCs in KBM ADSC-4 under hypoxic conditions, EVs secreted from hDPSCs contained angiogenic miRNAs. To identify angiogenic miRNAs, as the first screening, we isolated EVs from the conditioned medium under normoxic conditions and hypoxic conditions and then analyzed the expression levels of miRNAs encapsulated in EVs by array analysis (Fig. 2 a). The expression levels of miRNA numbers 1 to 5 were higher by more than fourfold in hypoxic conditions than in normoxic conditions (Table 1), and their expression was only detected under hypoxic conditions. The expression levels of miRNA numbers 6 to 13 were higher by >2.5-fold in hypoxic conditions than in normoxic conditions (Table 1). Although detected under normoxic conditions, their expression was upregulated under hypoxic conditions. In the second screening of identifying angiogenic miRNAs, we evaluated the proliferation of human aortic endothelial cells (HAoEc) after adding EVs containing each of the 12 miRNAs, excluding miRNA-1973 (number 8) (Fig. 2 b). EVs including miR-4485-3p, miR-765, or miR-4284 proliferated HAoEc. Although there was no statistically significant difference in miR-3678-3p, cell proliferation was at the same level as miR-765. So, miR-3678-3p was employed for further analysis. The third screening, we injected EVs containing four miRNAs into the left femur of the mouse hindlimb ischemia model and then examined the blood flow (Fig. 2 c). The blood flow was significantly higher in EVs containing miR-765 than that in the control.

**Table 1:** The expression levels of miRNA in extracellular vesicles (EVs) by array analysis.

Number	miRNA name	Ratio (Hypoxic/Normoxic)	Globalization	
			Normoxic	Hypoxic
1	hsa-miR–889-3p	11.64	0.00	11.29
2	hsa-miR–659-5p	7.31	0.00	7.09
3	hsa-miR-4519	5.17	0.00	5.01
4	hsa-miR-551b-5p	4.06	0.00	3.94
5	hsa-miR–7853-5p	4.05	0.00	3.93
6	hsa-miR–4485-3p	3.61	43.61	152.79
7	hsa-miR-765	3.39	137.35	451.45
8	hsa-miR-1973	3.06	109.00	324.09
9	hsa-miR-6769b-5p	2.97	381.95	1098.75
10	hsa-miR-4284	2.94	324.06	923.04
11	hsa-miR–3678-3p	2.81	67.29	183.61
12	hsa-miR–4756-5p	2.72	198.66	524.45
13	hsa-miR–3162-5p	2.59	393.28	988.90

### Characterization of miR-765–encapsulated EVs

**Fig. 3:**
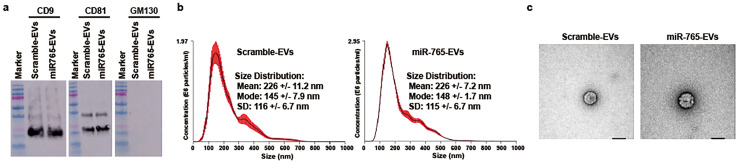
Characteristics of extracellular vesicles (EVs) secreted from 293 T cells. These cells were incubated with HE100 medium supplemented with L-glutamine, and transfected with microRNA (miRNA) mimics. The conditioned medium was concentrated using Amicon Ultra-15, and miRNA mimic–encapsulated EVs were isolated using qEV column for concentration solution. a: EV marker analysis. Western blotting analysis was conducted using specific antibodies for isolated EVs. CD9 and CD81 were EV-positive markers, whereas GM130 (a Golgi apparatus marker) was an EV-negative marker. b: Size distribution analysis of isolated EVs. Isolated EVs were examined by nanoparticle tracking analysis. Brownian motion was filmed 5 times. Red error bars indicate +/−1 standard error of the mean. c: Morphological analysis of isolated EVs. Isolated EVs were lysed using a negative-staining transmission electron micrograph. The black bar indicates 100 nm.

To analyze the characteristics of the miRNA mimic–containing EVs, we conducted three kinds of experiments (Fig. 3). Both scramble- and miR-765–encapsulated EVs had CD9 and CD81 (EV markers) but had no GM130 (a Golgi apparatus marker) (Fig. 3 a). The mean size was 226 ± 11.2 and 226 ± 7.2 nm in scramble- and miR-765–encapsulated EVs, respectively (Fig. 3 b). Regarding morphology, EVs were round shaped (Fig. 3 c).

### miR-765–encapsulated EVs increase the mRNA expression levels of angiogenic genes in the BALB/c mice ischemia model

**Fig. 4:**
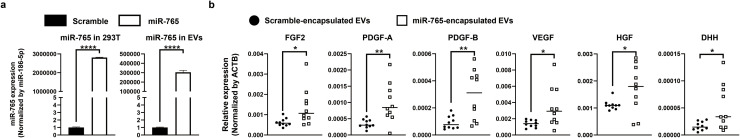
The mRNA expression analysis of growth factors in the mouse hindlimb ischemia model after injecting microRNA (miRNA) mimic–encapsulated extracellular vesicles (EVs). a: Expression level of miR-765 analyzed using quantitative polymerase chain reaction (qPCR) in 293 T cells transfected with miRNA mimics (left) and EVs secreted from 293 T cells transfected with miRNA mimics (right) (n = 3/group; unpaired *t*-test). b: Expression levels of angiogenic mRNAs analyzed using qPCR. A mouse hindlimb ischemia model was injected with miRNA mimic–encapsulated EVs. Total RNAs were extracted from thigh tissue 2 days after EV injection (n = 10/group; unpaired *t*-test). DHH, desert hedgehog; FGF-2, fibroblast growth factor 2; HGF, hepatocyte growth factor; PDGF-A, platelet-derived growth factor (PDGF) with A subunit; PDGF-B, PDGF with B subunit; VEGF, vascular endothelial growth factor.

To elucidate the angiogenic effect through miR-765, we transfected this miRNA into 293 T cells (Fig. 4 a left) and manufactured miR-765–encapsulated EVs (Fig. 4 a [right]). The inclusion amount of miR-765 was approximately 300,000-fold in miR-765–encapsulated EVs compared with that in scramble-encapsulated EVs. At 2 days after injecting miR-765–encapsulated EVs in the left femur of the mouse hindlimb ischemia, we analyzed the expression levels of angiogenic miRNAs. The expression levels of FGF2, PDGF-A, PDGF-B, VEGF, HGF, and desert hedgehog (DHH) were significantly higher in miR-765–encapsulated EVs than in scramble-encapsulated EVs (Fig. 4 b).

### miR-765 targets DPP4 and increases FGF2 expression

**Fig. 5:**
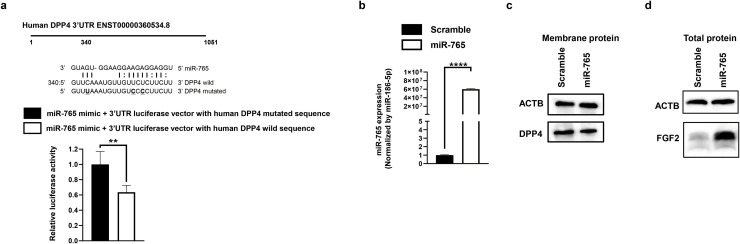
Fibroblast growth factor 2 (FGF2) upregulation after miR-765 bound to the 3′ untranslated region (3′UTR) of human dipeptidyl peptidase 4 (DPP4). a: Human DPP4 3′UTR sequences (ENST00000360534.8) and complementary miR-765–binding sequences. The upper, middle, and lower sequences represent miR-765, the target wild-type of DPP4, and the target mutated sequence of DPP4, respectively. The underbars denote the mutated sites. In the 3′UTR luciferase assay, 293 T cells were transfected with miR-765 mimic and 3′UTR vectors with wild- or mutated-type sequence. Relative luciferase activities of miR-765 mimic and 3′UTR vectors with the wild-type sequence were normalized using miR-765 mimic and 3′UTR vectors with the mutated-type sequence, presented as 1 (The sample size was 4 in each group; unpaired t-test). b: Expression level of miR-765 in 293 T cells. The miR-765 mimics were transfected into 293 T cells. The miR-765 expression level was confirmed using qPCR at 2 days after transfection. c: DPP4 protein level in 293 T cells transfected with miR-765. Membrane protein was extracted at 3 days after transfection. DPP4 protein level was analyzed by western blotting. Actin beta (ACTB) was used as an internal control. d: FGF2 protein level in 293 T cells transfected with miR-765. Total protein was extracted at 3 days after transfection. FGF2 protein level was analyzed by western blotting. ACTB was used as an internal control.

The target genes of miR-765 were identified through 3′ untranslated region (3′UTR) luciferase reporter assay. We predicted that miR-765 targeted one site (3′UTR: 340) within the human DPP4 3′UTR sequence. The assay showed that miR-765 targeted the human DPP4 3′UTR sequence of 340 (Fig. 5 a). We also predicted that miR-765 targeted eight potential complimentary binding sites of the mouse DPP4 3′UTR sequence based on an algorism (Supplementary Fig. 2). However, the assay revealed that miR-765 only targeted one site (3′UTR: 1707) within the mouse DPP4 3′UTR sequence (Supplementary Fig. 2). To examine the inhibitory effect of miR-765 on the protein level, we conducted western blotting analysis at 3 days after miR-765 mimic transfection into 293 T cells. The high expression level of miR-765 was recognized in miR-765 mimic–transfected cells compared with that in scramble mimic–transfected cells (Fig. 5 b). The protein level of DPP4 was decreased in miR-765 mimic–transfected cells (Fig. 5 c) but FGF2 protein levels were increased in miR-765 mimic–transfected cells (Fig. 5 d). We designed shRNAs for DPP4 and performed DPP4-knockdown experiment in 293 T cells. The 293 T cells, HAoECs, and HSMMs were transfected with shRNAs targeting DPP4. Expression levels of DPP4 mRNAs were decreased in DPP4-knockdowned 293 T cells as compared to controls, while FGF2 protein levels were increased (Supplementary Figure 6). However, DPP4 protein levels were decreased in DPP4-knockdowned HAoECs as compared to controls, while FGF2 protein levels were not increased in DPP4-knockdown HAoECs (Supplementary Figure 7). The mouse hindlimb model of ischemia was constructed by injection of EVs carrying miR-765 into the thigh, which increased the mRNA levels of FGF2. Therefore, HSMMs were used as thigh-derived cells (Supplementary Figure 8). Protein levels of DPP4 were decreased in DPP4-knockdown HSMMs as compared to controls, while FGF2 protein levels were increased.

## Discussion

EVs obtained from conditioned medium reportedly have no immunogenicity [33]. Allogenic cells, excluding pluripotent stem cells and somatic cells, can be derived from the bone marrow, adipose tissue, umbilical cord blood, and dental pulp. Young and healthy individuals are preferable donors of allogenic cells, which can be isolated with non-invasive procedures. Therefore, we excluded BM-MSCs and Ad-MSCs from allogenic cells. DPSCs have a slightly higher angiogenic capacity than UC-MSCs [34]. Therefore, DPSCs were selected as allogenic cells for isolation of EVs.

This study used hDPSCs as cells that secreted EVs to explore angiogenic miRNAs because these cells were established from young and healthy donors. EVs secreted from hDPSCs contained VEGF, HGF, angiopoietin-1, FGF2, interleukin 4 (IL-4), and PDGF-BB (Fig. 1 a) and enhanced the blood flow of the mouse hindlimb ischemia model (Fig. 1 b). However, improving the blood flow of the mice hindlimb ischemia model after injecting EVs secreted from hDPSCs remains poorly studied. We first applied hypoxic conditions to rat bone marrow cells [35], with reference to the culture temperature of 33°C used for haemopoietic stem cells [36]. We reported that hypoxic preconditioning enhances angiogenesis after transplanting cells in ischemic tissues [37,38]. To recognize whether hypoxic culture affects EVs, we cultured hDPSCs for 7 days under a serum-free medium. Hypoxic culture inhibited cell proliferation, but the concentration of VEGF involved in EVs increased in hypoxic conditions compared with that in normoxic conditions (Fig. 1 c and Supplementary Figure 5). Thus, angiogenic miRNAs might increase in EVs secreted from hDPSCs under hypoxic conditions. To identify angiogenic miRNAs in EVs secreted from hDPSCs, as the first screening, we conducted a miRNA array analysis using miRNAs isolated from EVs in a medium under normoxic or hypoxic conditions (Fig. 2 a). We chose 13 miRNAs as angiogenic miRNA candidates according to the comparative expression analysis (Table 1). However, we did not find the genome sequence information of miRNA-1973 (number 8) in the database at that time; hence, only 12 miRNAs moved on to the second screening. Cell proliferation significantly increased after injecting EVs including miR-4485-3p, miR-765, or miR-4284 compared with the control (Fig. 2 b). These three miRNAs and miR-3678-3p proceeded to the third screening. We found that EVs including miRNA-765 significantly improved the blood flow in the mouse hindlimb ischemia model compared with the control (Fig. 2 c). Therefore, miR-765 was considered as an angiogenic miRNA.

Some papers, particularly cancer research, have reported the function of miR-765. This miRNA was downregulated in hypoxia-induced 3D channel–like structures of SKOV3 ovarian cancer cells, and its low expression was associated with poor overall survival because it targeted the 3′UTR of VEGF-A [39]. In addition, the negative expression of miR-765 was associated with poor overall survival in osteosarcoma, while miR-765 expression was inversely related to VEGF expression [40]. These reports do not support our data because hDPSCs cultured under hypoxic conditions secreted EVs containing high concentrations of VEGF and miR-765 compared with those cultured under normoxic conditions (Supplementary Figure 5 and Table 1). Although miR-765 was downregulated in both mouse brains after cerebral ischemia/reperfusion and neuroblastoma cells under hypoxic culture, the miR-765 mimic showed an antiapoptotic effect by targeting the 3′UTR of BCL2L13 [41] Although miR-765 and VEGF were upregulated in EVs secreted from hDPSCs under hypoxic conditions (Table 1 and Supplementary Figure 5), this result may be a characteristic of hDPSCs similar to the characteristics of mesenchymal stem cells [42].

In this study, miRNAs were encapsulated in EVs originating from 293 T cells and applied to cells and tissues (Fig. 2, Fig. 4 b). The 293 T cells can be cultured using a serum-free medium that is commercially available for protein production. Thus, miR-765 mimics were transfected into 293 T cells, which were cultured using HE100 medium supplemented with L-glutamine to obtain miR-765–encapsulated EVs. Isolated EVs including miR-765 were characterized in three ways [43]. In EVs, CD9 and CD81 were detected as EV-positive markers, but GM130 was not detected as a Golgi apparatus marker and an EV-negative marker (Fig. 3 a). The mean diameter was 226 nm in both isolated miR-765- and scramble-encapsulated EVs (Fig. 3 b), and their shape was round (Fig. 3 c). The inclusion amount of miR-765 in EVs was approximately 300,000-fold higher than that of scramble (Fig. 4 a). The miR-765–encapsulated EVs upregulated the mRNA expression levels of angiogenic factors compared with the scramble-encapsulated EVs at 2 days after injection in thigh tissues of the mouse hindlimb ischemia model (Fig. 4 b) [[44], [45], [46], [47]].

DPP4 knockdown increases FGF2 protein in lymph node carcinoma of the prostate (LNCaP) cells [48]. Therefore, to investigate whether miR-765 inhibited DPP4, we conducted a 3′UTR luciferase assay and western blot analysis. The relationship between DPP4 and miR-765 was investigated using RNA22 v2 miRNA target detection software, which has a lot of flexibility to analyze RNA-RNA base pairing. This software can analyze the relationship between microRNAs and genes from different species, such as human microRNAs and mouse genes. Although microRNA binding typically occurs in the seed region, the non-seed region also effects microRNA binding to 3′UTR of target genes [49]. Notably, the non-seed region of miR-765 targets the 3′UTR of multiple genes [[50], [51], [52]], including DPP4. Our data showed that miR-765 targeted human and mouse DPP4 3′UTR sequences (Fig. 5 a, Fig. 5 c, and Supplementary Fig. 2) and increased FGF2 protein 3 days after transfection. DPP4 cleaves the N-terminal of FGF2 and represses angiogenesis induced by FGF2 protein in a mouse model [53]. Moreover, the administration of FGF2 can reduce ischemia of the lower limbs [54]. In the present study, injection of EVs carrying miR-765 increased the mRNA levels of FGF2 in the ischemic thigh tissues and induced angiogenesis (Fig. 4 b). Moreover, exogenous FGF2 induces endogenous FGF2 expression in pulmonary artery smooth muscle cells [55], indicating a positive feedback of FGF2. Although not increased in DPP4-knockdown HAoECs (Supplementary Figure 7), FGF2 protein levels were increased in DPP4-knockdown HSMMs (Supplementary Figure 8). Since Actin beta (ACTB) expression levels are highly variable in skeletal muscle myoblasts, α/β Tubulin was also used as internal control (Supplementary Figure 8). These results suggest that miR-765 increased FGF2 expression in skeletal muscle myoblasts of ischemic thigh tissues and induced angiogenesis. In conclusion, miR-765 demonstrated as an angiogenic miRNA in hDPSCs cultured under hypoxic condition. This miRNA exhibits an angiogenic effect by increasing FGF2 expression through DPP4 repression. Therefore, miR-765 may be used as an angiogenic therapeutic drug for patients with CLI.

## Declaration on generative AI and AI-assisted technologies in the writing process

The authors declare no use of Generative AI and AI-assisted technologies in the writing process during the preparation of this work.

## CRediT authorship contribution statement

**Koji Ueno:** Writing – review & editing, Writing – original draft, Project administration, Methodology, Investigation, Formal analysis, Conceptualization. **Hiroshi Kurazumi:** Writing – original draft, Methodology, Investigation, Formal analysis. **Junichi Murakami:** Investigation, Formal analysis. **Takahiro Mizoguchi:** Methodology. **Ryo Suzuki:** Investigation. **Toshiki Tanaka:** Formal analysis. **Kimikazu Hamano:** Writing – review & editing, Writing – original draft, Supervision.

## Declaration of competing interest

The authors declare no competing financial interests.

## Data Availability

Data will be made available on request.

## Appendix Supplementary materials

Image, application 1

Image, application 2

Image, application 3

Image, application 4

Image, application 5

Image, application 6

Image, application 7

Image, application 8

Image, application 9

## References

[1] Fereydooni A., Gorecka J., Dardik A. (2020). Using the epidemiology of critical limb ischemia to estimate the number of patients amenable to endovascular therapy. Vasc. Med..

[2] Hassanshahi M. (2019). Critical limb ischemia: current and novel therapeutic strategies. J. Cell Physiol..

[3] Schorr E.N., Treat-Jacobson D. (2013). Methods of symptom evaluation and their impact on peripheral artery disease (PAD) symptom prevalence: a review. Vasc. Med..

[4] Qadura M., Terenzi D.C., Verma S., Al-Omran M., Hess D.A. (2018). Concise review: cell therapy for critical limb ischemia: an integrated review of preclinical and clinical studies. Stem Cell..

[5] Dormandy J., Heeck L., Vig S. (1999). The fate of patients with critical leg ischemia. Semin. Vasc. Surg..

[6] Norgren L. (2007). Inter-society consensus for the management of peripheral arterial disease (TASC II). J. Vasc. Surg..

[7] Hirsch A.T. (2006). ACC/AHA. 2005 guidelines for the management of patients with peripheral arterial disease (lower extremity, renal, mesenteric, and abdominal aortic): executive summary a collaborative report from the American association for vascular surgery/society for vascular surgery, society for cardiovascular angiography and interventions, society for vascular medicine and biology. Society of interventional radiology, and the ACC/AHA task force on practice guidelines (writing committee to develop guidelines for the management of patients with peripheral arterial disease) endorsed by the American association of cardiovascular and pulmonary rehabilitation; national heart, lung, and blood institute; society for vascular nursing; transatlantic inter-society consensus; and vascular disease foundation. J. Am. Coll. Cardiol..

[8] Lawall H., Bramlage P., Amann B. (2011). Treatment of peripheral arterial disease using stem and progenitor cell therapy. J. Vasc. Surg..

[9] Powell R.J. (2012). Update on clinical trials evaluating the effect of biologic therapy in patients with critical limb ischemia. J. Vasc. Surg..

[10] Han J., Luo L., Marcelina O., Kasim V., Wu S. (2022). Therapeutic angiogenesis-based strategy for peripheral artery disease. Theranostics.

[11] Esato K. (2002). Neovascularization induced by autologous bone marrow cell implantation in peripheral arterial disease. Cell Transpl..

[12] Kudo T. (2014). Hypoxic preconditioning reinforces cellular functions of autologous peripheral blood-derived cells in rabbit hindlimb ischemia model. Biochem. Biophys. Res. Commun..

[13] Kudo T. (2014). Hypoxically preconditioned human peripheral blood mononuclear cells improve blood flow in hindlimb ischemia xenograft model. Am. J. Transl. Res..

[14] Samura M. (2016). Combinatorial treatment with apelin-13 enhances the therapeutic efficacy of a preconditioned cell-based therapy for peripheral ischemia. Sci. Rep..

[15] Samura M. (2017). Therapeutic strategies for cell-based neovascularization in critical limb ischemia. J. Transl. Med..

[16] Sahoo S. (2011). Exosomes from human CD34(+) stem cells mediate their proangiogenic paracrine activity. Circ. Res..

[17] Mathiyalagan P. (2017). Angiogenic mechanisms of human CD34+ stem cell exosomes in the repair of ischemic hindlimb. Circ. Res..

[18] Ye M. (2019). Exosomes derived from human induced pluripotent stem cells-endothelia cells promotes postnatal angiogenesis in mice bearing ischemic limbs. Int. J. Biol. Sci..

[19] Zhang Y. (2020). Knockout of beta-2 microglobulin reduces stem cell-induced immune rejection and enhances ischaemic hindlimb repair via exosome/miR-24/bim pathway. J. Cell Mol. Med..

[20] Qiu X. (2022). Prophylactic exercise-derived circulating exosomal miR-125a-5p promotes endogenous revascularization after hindlimb ischemia by targeting endothelin converting enzyme 1. Front. Cardiovasc. Med..

[21] Hu G.W. (2015). Exosomes secreted by human-induced pluripotent stem cell-derived mesenchymal stem cells attenuate limb ischemia by promoting angiogenesis in mice. Stem Cell Res. Ther..

[22] Zhu Q. (2018). Extracellular vesicles secreted by human urine-derived stem cells promote ischemia repair in a mouse model of hind-limb ischemia. Cell Physiol. Biochem..

[23] Zhu D. (2020). Macrophage M2 polarization induced by exosomes from adipose-derived stem cells contributes to the exosomal proangiogenic effect on mouse ischemic hindlimb. Stem Cell Res. Ther..

[24] Zhang X. (2021). Exosomes derived from adipose-derived stem cells overexpressing glyoxalase-1 protect endothelial cells and enhance angiogenesis in type 2 diabetic mice with limb ischemia. Stem Cell Res. Ther..

[25] Srivastava A. (2020). Progress in extracellular vesicle biology and their application in cancer medicine. Wiley. Interdiscip. Rev. Nanomed. Nanobiotechnol..

[26] Théry C. (2018). Minimal information for studies of extracellular vesicles 2018 (MISEV2018): a position statement of the international society for extracellular vesicles and update of the MISEV2014 guidelines. J. ExtraCell Vesicles..

[27] Ueno K. (2016). Treatment of refractory cutaneous ulcers with mixed sheets consisting of peripheral blood mononuclear cells and fibroblasts. Sci. Rep..

[28] Tanaka Y. (2016). Autologous preconditioned mesenchymal stem cell sheets improve left ventricular function in a rabbit old myocardial infarction model. Am. J. Transl. Res..

[29] Takeuchi Y. (2017). Ulcer healing effect of autologous mixed sheets consisting of fibroblasts and peripheral blood mononuclear cells in rabbit ischemic hind limb. Am. J. Transl. Res..

[30] Fujita A. (2019). Hypoxic-conditioned cardiosphere-derived cell sheet transplantation for chronic myocardial infarction. Eur. J. Cardiothorac. Surg..

[31] Ueno K. (2011). Tumour suppressor microRNA-584 directly targets oncogene Rock-1 and decreases invasion ability in human clear cell renal cell carcinoma. Br. J. Cancer.

[32] Ueno K. (2024). miR-709 exerts an angiogenic effect through a FGF2 upregulation induced by GSK3B downregulation. Sci. Rep..

[33] Zhu X. (2017). Comprehensive toxicity and immunogenicity studies reveal minimal effects in mice following sustained dosing of extracellular vesicles derived from HEK293T cells. J. ExtraCell Vesicles..

[34] Angelopoulos I. (2022). Delivery of affordable and scalable encapsulated allogenic/autologous mesenchymal stem cells in coagulated platelet poor plasma for dental pulp regeneration. Sci. Rep..

[35] Li T.S. (2002). Improved angiogenic potency by implantation of ex vivo hypoxia prestimulated bone marrow cells in rats. Am. J. Physiol. Heart. Circ. Physiol..

[36] Dexter T.M., Allen T.D., Lajtha L.G (1977). Conditions controlling the proliferation of haemopoietic stem cells in vitro. J. Cell Physiol..

[37] Kubo M. (2008). Hypoxic preconditioning increases survival and angiogenic potency of peripheral blood mononuclear cells via oxidative stress resistance. Am. J. Physiol. Heart. Circ. Physiol..

[38] Kubo M. (2009). Increased expression of CXCR4 and integrin alphaM in hypoxia-preconditioned cells contributes to improved cell retention and angiogenic potency. J. Cell Physiol..

[39] Salinas-Vera Y.M. (2019). HypoxamiRs profiling identify miR-765 as a regulator of the early stages of vasculogenic mimicry in SKOV3 ovarian cancer cells. Front. Oncol..

[40] Liang W. (2017). MicroRNA-765 enhances the anti-angiogenic effect of CDDP via APE1 in osteosarcoma. J. Cancer.

[41] Lu Y. (2020). LncRNA FOXD3-AS1 knockdown protects against cerebral ischemia/reperfusion injury via miR-765/BCL2L13 axis. Biomed. PharmacOther.

[42] Bar J.K., Lis-Nawara A., Grelewski P.G. (2021). Dental pulp stem cell-derived secretome and its regenerative potential. Int. J. Mol. Sci..

[43] Lötvall J. (2014). Minimal experimental requirements for definition of extracellular vesicles and their functions: a position statement from the International Society for extracellular vesicles. J. ExtraCell Vesicles..

[44] Shoeibi S., Mozdziak P., Mohammadi S. (2018). Important signals regulating coronary artery angiogenesis. Microvasc. Res..

[45] Shirbaghaee Z., Hassani M., Heidari Keshel S., Soleimani M. (2022). Emerging roles of mesenchymal stem cell therapy in patients with critical limb ischemia. Stem Cell Res. Ther..

[46] Mabotuwana N.S. (2022). Paracrine factors released by stem cells of mesenchymal origin and their effects in cardiovascular disease: a systematic review of pre-clinical studies. Stem Cell Rev. Rep..

[47] Renault M.A. (2013). Desert hedgehog promotes ischemia-induced angiogenesis by ensuring peripheral nerve survival. Circ. Res..

[48] Wesley U.V., McGroarty M., Homoyouni A. (2005). Dipeptidyl peptidase inhibits malignant phenotype of prostate cancer cells by blocking basic fibroblast growth factor signaling pathway. Cancer Res..

[49] Duan Y. (2022). Critical contribution of 3′ non-seed base pairing to the in vivo function of the evolutionarily conserved let-7a microRNA. Cell Rep..

[50] Liang W. (2019). MicroRNA-765 sensitizes osteosarcoma cells to cisplatin via downregulating APE1 expression. Onco Targets. Ther..

[51] Qian C.J. (2020). LncRNA MAFG-AS1 accelerates cell migration, invasion and aerobic glycolysis of esophageal squamous cell carcinoma cells via miR-765/PDX1 axis. Cancer Manage Res..

[52] Seok H.J. (2024). miR-765 as a promising biomarker for low-dose radiation-induced pulmonary fibrosis. Noncoding. RNA Res..

[53] Suda M. (2017). Inhibition of dipeptidyl peptidase-4 ameliorates cardiac ischemia and systolic dysfunction by up-regulating the FGF-2/EGR-1 pathway. PLoS. One.

[54] Baffour R. (1992). Enhanced angiogenesis and growth of collaterals by in vivo administration of recombinant basic fibroblast growth factor in a rabbit model of acute lower limb ischemia: dose-response effect of basic fibroblast growth factor. J. Vasc. Surg..

[55] Black S.M., DeVol J.M., Wedgwood S. (2008). Regulation of fibroblast growth factor-2 expression in pulmonary arterial smooth muscle cells involves increased reactive oxygen species generation. Am. J. Physiol. Cell Physiol..

