# Supplementary figures and images for: miR-765 induces angiogenesis by inhibiting dipeptidyl peptidase 4 and increasing fibroblast growth factor 2
Source: BBA Adv. 2026 Apr 29;9:100193. doi: 10.1016/j.bbadva.2026.100193 (PMC13158388; doi:10.1016/j.bbadva.2026.100193)

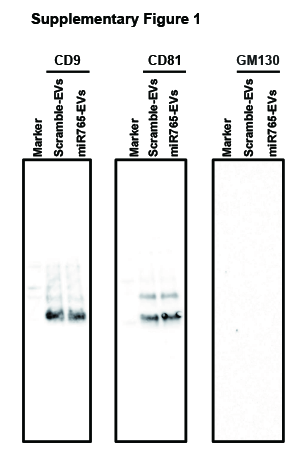

Supplement: Supplementary file 1 [file mmc1.zip › Supplementary Figure 1 miR-765.tif]

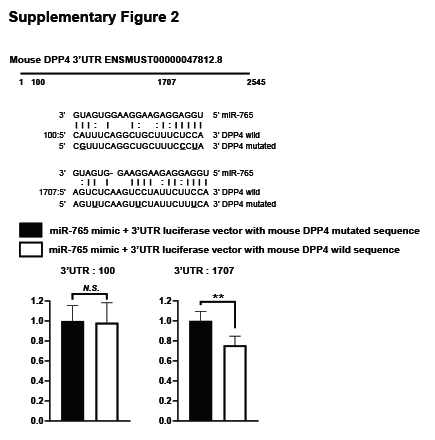

Supplement: Supplementary file 2 [file mmc2.zip › Supplementary Figure 2 miR-765.tif]

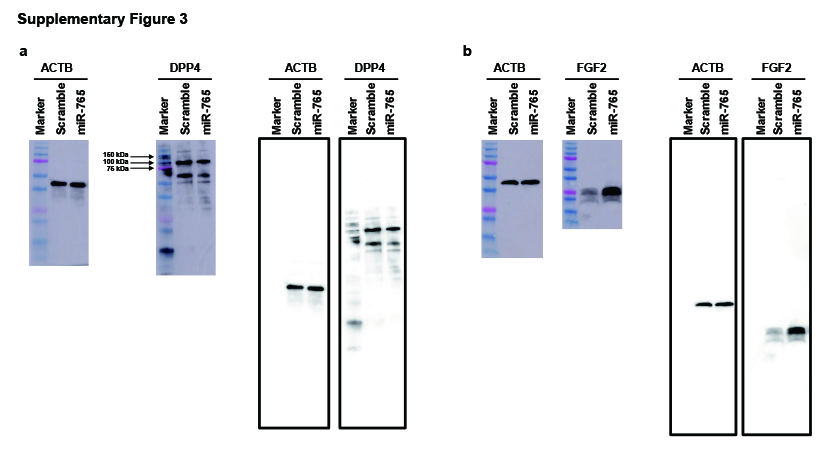

Supplement: Supplementary file 3 [file mmc3.zip › Supplementary Figure 3 miR-765.tif]

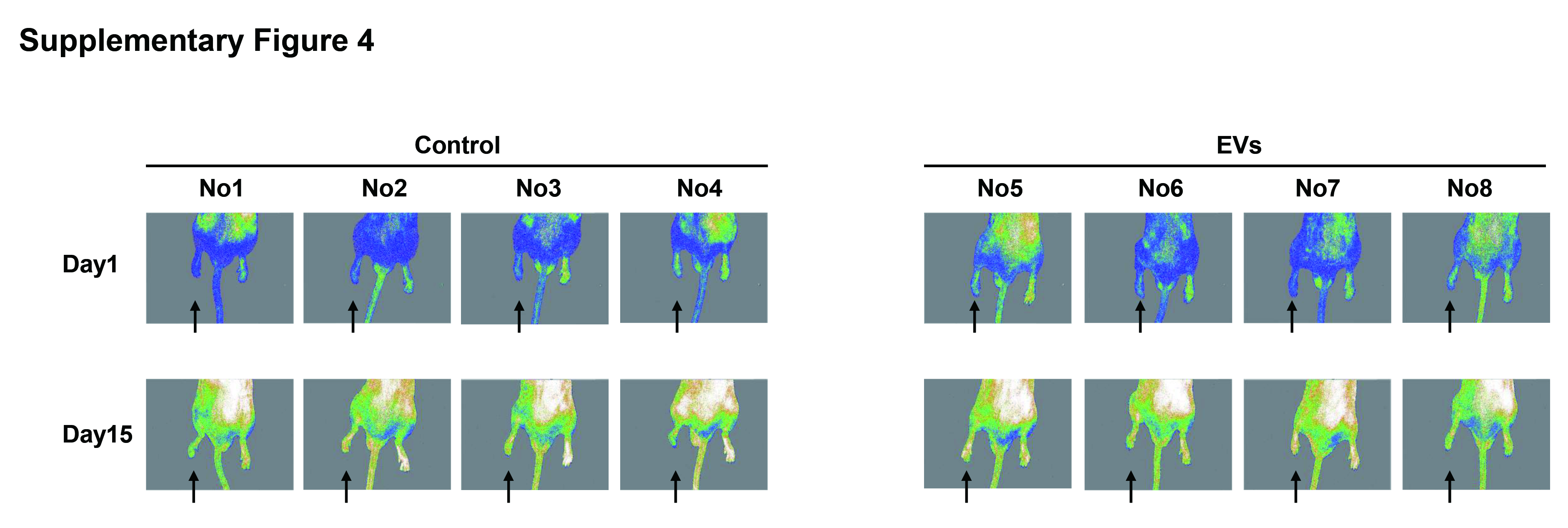

Supplement: Supplementary file 4 [file mmc4.zip › Supplementary Figure 4 miR-765 BBA Advance revised.tif]

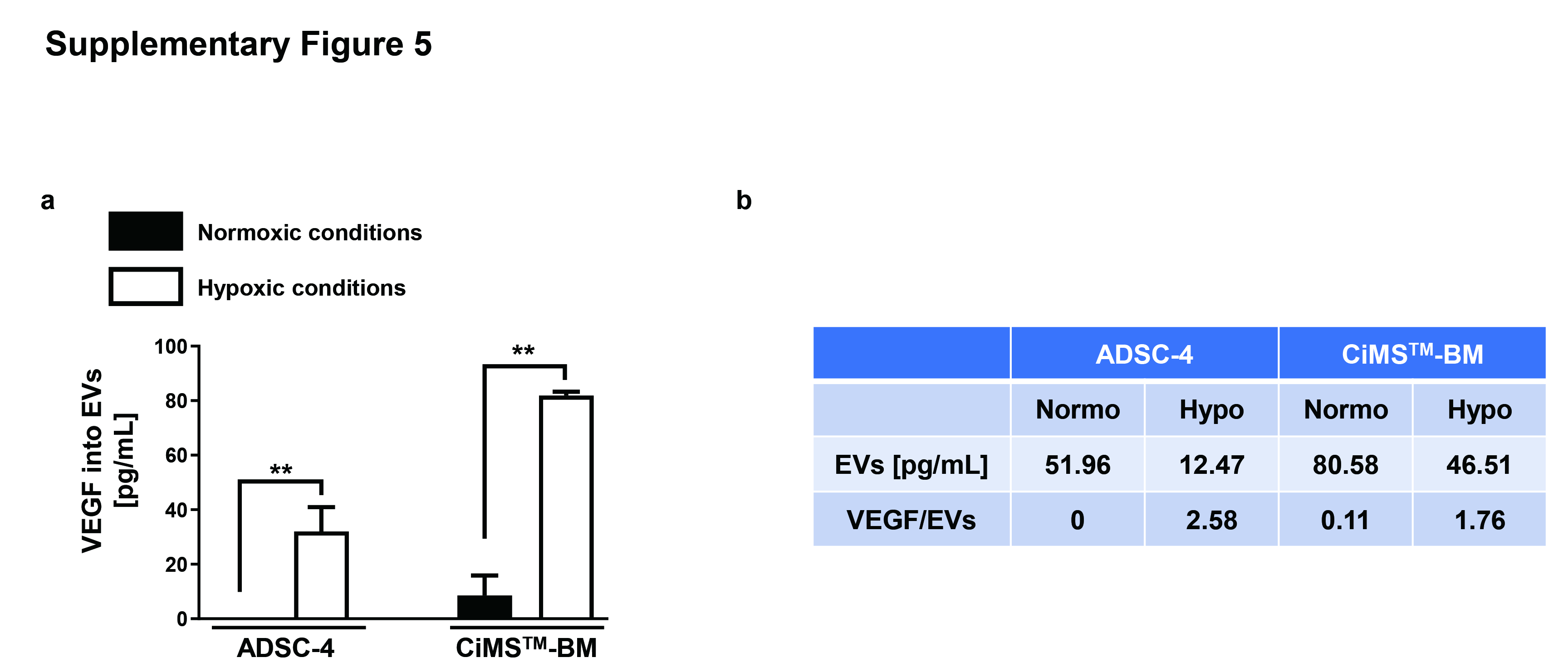

Supplement: Supplementary file 5 [file mmc5.zip › Supplementary Figure 5 miR-765 BBA Advance revised.tif]

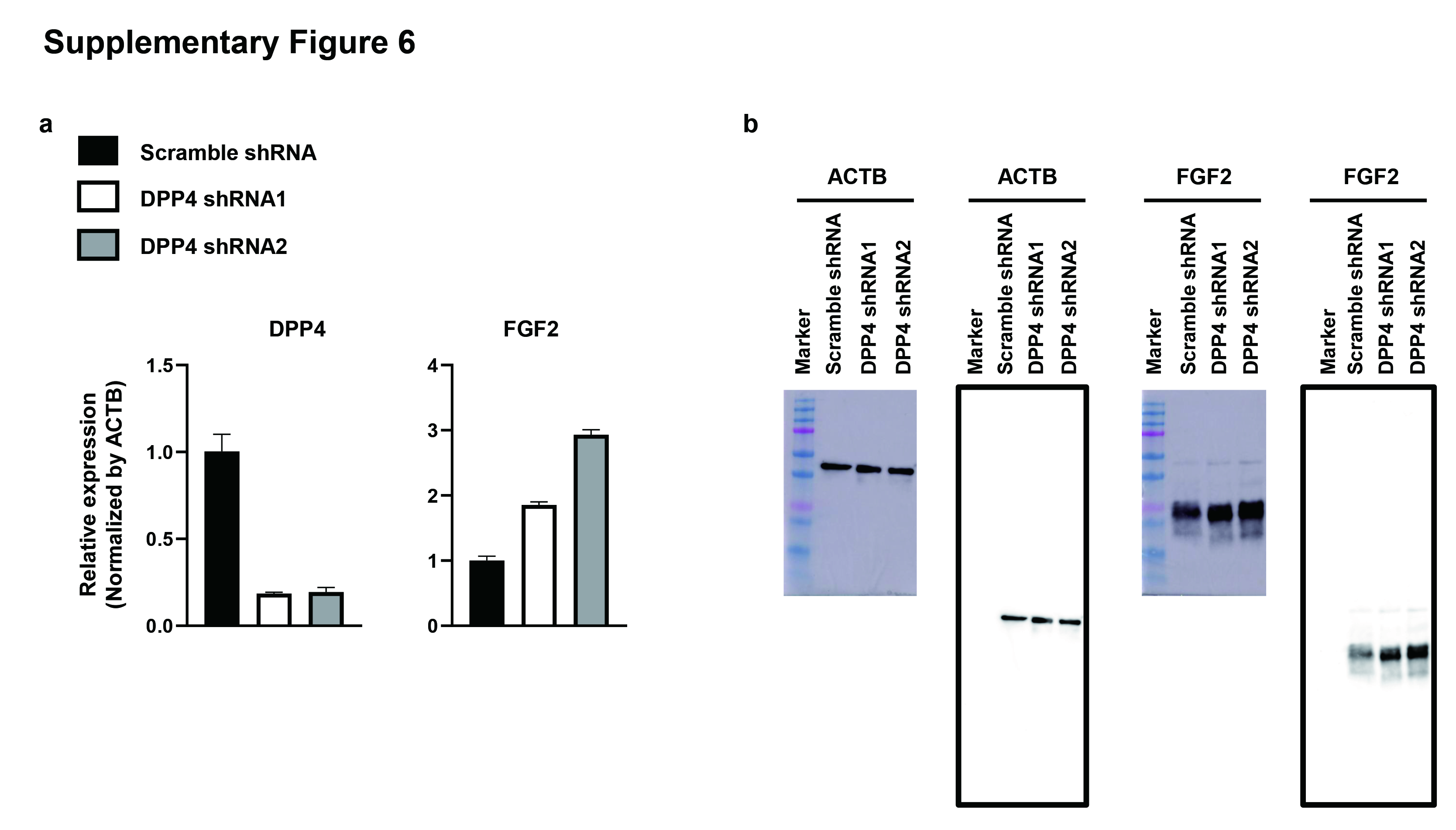

Supplement: Supplementary file 6 [file mmc6.zip › Supplementary Figure 6 miR-765 BBA Advance revised.tif]

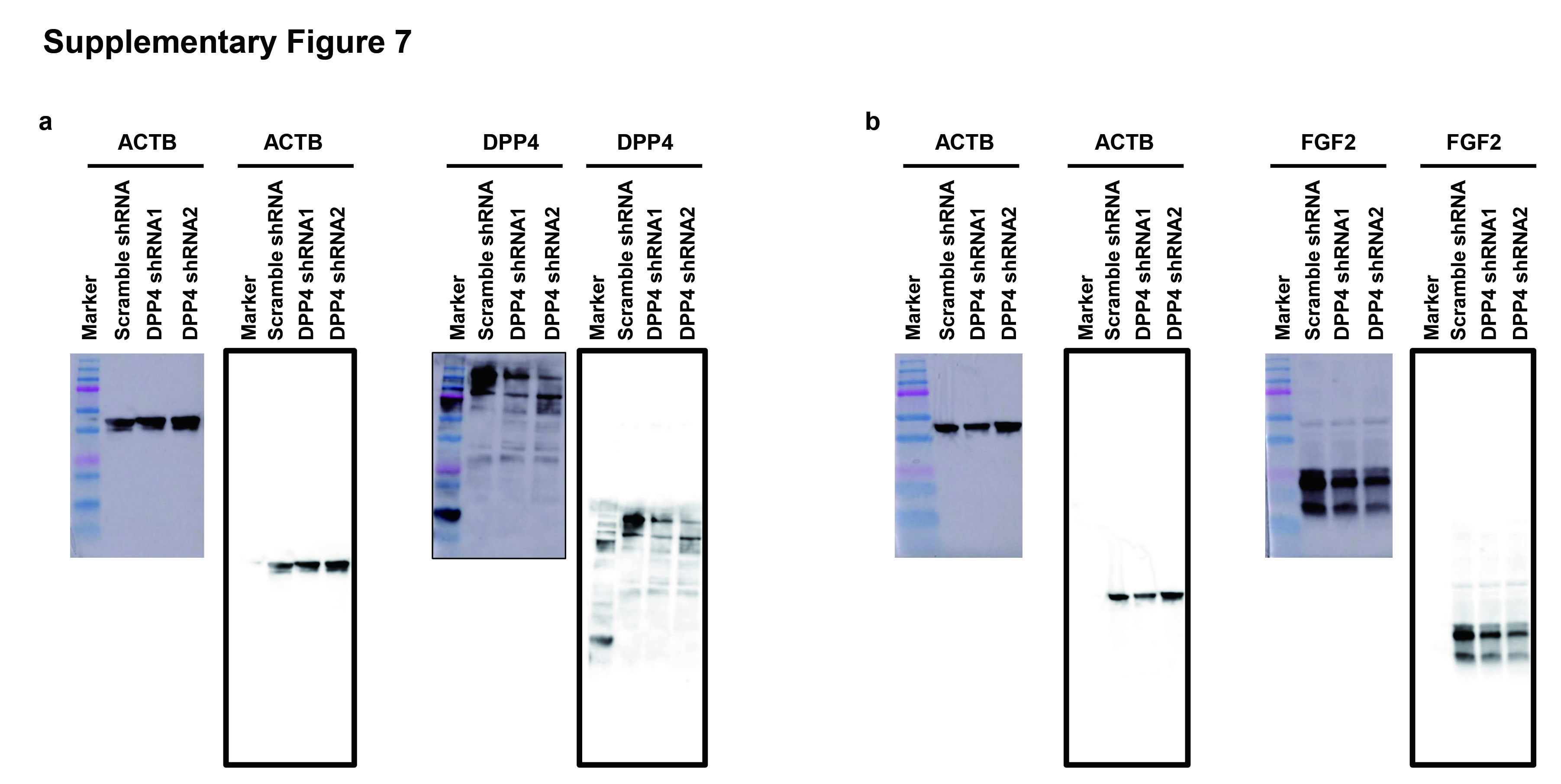

Supplement: Supplementary file 7 [file mmc7.zip › Supplementary Figure 7 miR-765 BBA Advance revised.tif]

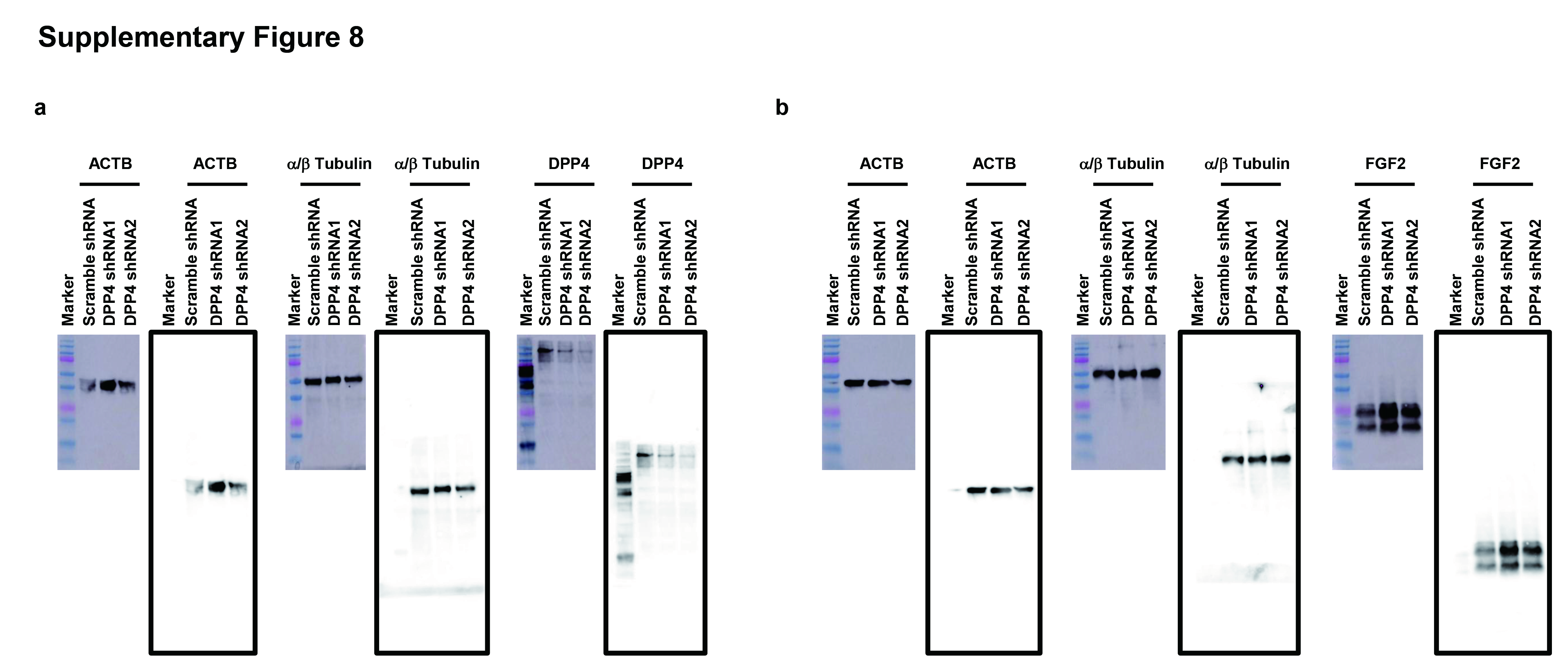

Supplement: Supplementary file 8 [file mmc8.zip › Supplementary Figure 8 miR-765 BBA Advance revised.tif]
