# Supplementary material for: miR-765 induces angiogenesis by inhibiting dipeptidyl peptidase 4 and increasing fibroblast growth factor 2
Source: BBA Adv. 2026 Apr 29;9:100193. doi: 10.1016/j.bbadva.2026.100193 (PMC13158388; doi:10.1016/j.bbadva.2026.100193)
Supplement: Supplementary file 9 [file mmc9.zip › Revised BBA Supplementary Table miR-765.pdf]

**miR-765 induces angiogenesis by inhibiting dipeptidyl peptidase 4 and increasing fibroblast growth factor 2**

Koji Ueno[1,2]*, Hiroshi Kurazumi[1], Junichi Murakami[1,2], Takahiro Mizoguchi[1], Ryo Suzuki[1], Toshiki Tanaka[1], and Kimikazu Hamano[1,2]

[1] Department of Surgery and Clinical Science, Graduate School of Medicine, Yamaguchi University, Ube, Yamaguchi, Japan

[2] Division of Advanced Cell Therapy, Research Institute for Cell Design Medical Science, Yamaguchi University, Ube, Yamaguchi, Japan

***Address correspondence to:** Dr. Koji Ueno, Assistant Professor, Department of Surgery and Clinical Science, Yamaguchi University Graduate School of Medicine, Minami-kogushi 1-1-1, Ube, Yamaguchi 755-8505, Japan. Tel: +81-836-22-2261; Fax: +81-836-2423; E-mail: kjueno@yamaguchi-u.ac.jp

**Supplementary Table 1.** Sequences of miRNAs identified by the first screening

| Number | miRNA<br>accession number<br>chromosome range | Primer sequence 5′ → 3′ | Enzyme | PCR<br>product |
|---|---|---|---|---|
| 1 | hsa-miR-889-3p<br>MIMAT0004921<br>chr14:101047601-101048279 | CTCGAGatcctcattgtttggactacctgag<br>GGATCCaatcatcaacacagtcacactccat | XhoI<br>BamHI | 268 |
| 2 | hsa-miR-659-5p<br>MIMAT0022710<br>chr22:37847378-37848074 | CTCGAGcgttcttgttttgtgtttcttcctt<br>GAATTCgaagatgagggttggttagatcaga | XhoI<br>EcoRI | 291 |
| 3 | hsa-miR-4519<br>MIMAT0019056<br>chr16:30874966-30875623 | CTCGAGacccttccaacctttgtgacg<br>GAATTCctgactgcataacgggcatac | XhoI<br>EcoRI | 460 |
| 4 | hsa-miR-551b-5p<br>MIMAT0004794<br>chr3:168551554-168552249 | CTCGAGgctaactgtcctgacttcagacctc<br>GAATTCcatcaaatcattagctgtcaacctg | XhoI<br>EcoRI | 322 |
| 5 | hsa-miR-7853-5p<br>MIMAT0030428<br>chr6:6169004-6169735 | CTCGAGaatcaaaaagttctccagacattgc<br>GAATTCcatggctctatatcacaatcacctg | XhoI<br>EcoRI | 616 |
| 6 | hsa-miR-4485-3p<br>MIMAT0019019<br>chr11:10507970-10508626 | CTCGAGaaaagtaaaaggaactcggcaaatc<br>GAATTCgaaatttttcacgcaggtttgatag | XhoI<br>EcoRI | 350 |
| 7 | hsa-miR-765<br>MIMAT0003945<br>chr1:156935831-156936544 | CTCGAGaagacaaggcacatacctacctgac<br>GAATTCaggatttggtggtttgtacggttat | XhoI<br>EcoRI | 299 |
| 8 | hsa-miR-1973<br>MIMAT0009448 | | | |
| 9 | hsa-miR-6769b-5p<br>MIMAT0027620<br>chr1:206474503-206475164 | CTCGAGatcaggccacatgataacagagatt<br>GAATTCaaatggaatgattagcaggagtcag | XhoI<br>EcoRI | 566 |
| 10 | hsa-miR-4284<br>MIMAT0016915<br>chr7:73711017-73711697 | CTCGAGcacacccagctactttttgattttt<br>GAATTCattttctgtgtctgtagggagcttg | XhoI<br>EcoRI | 391 |
| 11 | hsa-miR-3678-3p<br>MIMAT0018103<br>chr17:75405769-75406462 | CTCGAGctcttcccccgtctaatcactttat<br>GGATCCttcctactcatcctccaagactcag | XhoI<br>BamHI | 469 |

| | | | | |
|---|---|---|---|---|
| 12 | hsa-miR-4756-5p<br>MIMAT0019899<br>chr20:54068108-54068785 | CTCGAGttgggcaagtaggactagtaaggtg<br>GAATTCttaattatgtttgcacaaacggatg | XhoI<br>EcoRI | 433 |
| 13 | hsa-miR-3162-5p<br>MIMAT0015036<br>chr11:59594777-59595458 | CTCGAGgctttatggactaacttgggaacct<br>GAATTCaaagcaagcatgtgtgtgtttatgt | XhoI<br>EcoRI | 342 |

Notes: Uppercase letters represent the restriction enzyme sites. Upper rows in each primer sequence represent the forward primers, and the lower rows represent the reverse primers. PCR, polymerase chain reaction; miRNA, microRNA.

**Supplementary Table 2.** Primer sequences used for the undertaking of quantitative PCR.

| Gene name | Forward primer 5′ → 3′ | Reverse primer 5′ → 3′ |
|---|---|---|
| mouse ACTB | ggtgtgatggtgggaatgg | tcaggatacctctcttgctctgg |
| mouse FGF2 | gcgacccacacgtcaaactac | gcacacactcccttgatagacac |
| mouse PDGF-A | gagatagactccgtaggggctgag | ctcgggcacatggttaatgg |
| mouse PDGF-B | cgtagatgaagatggggctgag | ctccctcgagatgagctttcc |
| mouse HGF | tgccctatttcccgttgtg | acccgcagttgttttgttttg |
| mouse VEGF | ccatgaactttctgctctcttgg | acttctgctctccttctgtcgtg |
| mouse DHH | cagaccgcctgatgacagag | tactccgggccacatgttc |

PCR, polymerase chain reaction. ACTB, actin beta; FGF2, fibroblast growth factor 2; PDGF-A, platelet-derived growth factor subunit A; PDGF-B, platelet-derived growth factor subunit B; HGF, hepatocyte growth factor; VEGF, vascular endothelial growth factor; DHH, desert hedgehog.

**Supplementary Table 3.** Oligo sequences used for constructing the 3′UTR plasmids for human DPP4.

| Plasmid's name | Oligo sequence 5′ → 3′ |
|---|---|
| 3′UTR 340 wild | AAACTAGCGGCCGCTAGTGTTCAAATGTTGTTCTCTTCTTT |
| | CTAGAAAGAAGAGAACAACATTTGAACACTAGCGGCCGCTAGTTT |
| 3′UTR 340 mutated | AAACTAGCGGCCGCTAGTGTTTAAATGTTGTCCCCTTCTTT |
| | CTAGAAAGAAGGGGACAACATTTAAACACTAGCGGCCGCTAGTTT |

Notes: Upper rows in each set of oligo sequences represent the forward oligo sequences, and the lower rows represent the reverse oligo sequences. 3′UTR, 3′ untranslated region; DPP4, dipeptidyl peptidase 4.

**Supplementary Table 4.** Oligo sequences used for constructing the 3′UTR plasmids for mouse DPP4.

| Plasmid's name | Oligo sequence 5′ → 3′ |
|---|---|

| | |
|---|---|
| 3′UTR 100 wild | AAACTAGCGGCCGCTAGTCATTTCAGGCTGCTTTCTCCAT |
| | CTAGATGGAGAAAGCAGCCTGAAATGACTAGCGGCCGCTAGTTT |
| 3′UTR 100 mutated | AAACTAGCGGCCGCTAGTCGTTTCAGGCTGCTTTCCCTAT |
| | CTAGATAGGGAAAGCAGCCTGAAACGACTAGCGGCCGCTAGTTT |
| 3′UTR 1707 wild | AAACTAGCGGCCGCTAGTAGTCTCAAGTCCTATTCTTCCAT |
| | CTAGATGGAAGAATAGGACTTGAGACTACTAGCGGCCGCTAGTTT |
| 3′UTR 1707 mutated | AAACTAGCGGCCGCTAGTAGTTTCAAGTTCTATTCTTTCAT |
| | CTAGATGAAAGAATAGAACTTGAAACTACTAGCGGCCGCTAGTTT |

Notes: Upper rows in each set of oligo sequences represent the forward oligo sequences, and the lower rows represent the reverse oligo sequences. 3′UTR, 3′ untranslated region; DPP4, dipeptidyl peptidase 4.

**Supplementary Table 5.** Primer sequences used for the undertaking of quantitative PCR.

| Gene name | Forward primer 5′ → 3′ | Reverse primer 5′ → 3′ |
|---|---|---|
| human ACTB | gctcctcctgagcgcaag | catctgctggaaggtggaca |
| human DPP4 | agcgaccctcacatcaagc | acggttagcacacactcctttg |
| Human FGF2 | tgcctactctgctctgtggtg | gggacttctgtgtcgttaaattgg |

**Supplementary Table 6** Target sequence and oligo sequences used for the construction of the plasmids.

| Name | Oligo sequence 5’ → 3’ |
|---|---|
| Scramble shRNA | GATCCgtgatactgttgtcagctgctgatcCTTCCTGTCAGAgatcagcagctgacaacagtatcacTTTTTG |
| | AATTCAAAAAgtgatactgttgtcagctgctgatcTCTGACAGGAAGgatcagcagctgacaacagtatcacG |
| DPP4 shRNA1 | GATCCtggacattctatcaatgattattCTTCCTGTCAGAaataatcattgatagaatgtccaTTTTTG |
| | AATTCAAAAAtggacattctatcaatgattattTCTGACAGGAAGaataatcattgatagaatgtccaG |
| DPP4 shRNA2 | GATCCggggaaagaagatataatatataCTTCCTGTCAGAtatatattatatcttctttccccTTTTTG |
| | AATTCAAAAAggggaaagaagatataatatataTCTGACAGGAAGtatatattatatcttctttccccG |

Notes: Upper rows in each oligo sequence set represent the forward oligo sequences, while the lower rows in each oligo sequence set represent the reverse oligo sequences. Underlined solid lines highlight the sense sequences of the target genes, while underlined dot lines highlight the antisense sequence of the target genes.
